# Rechargeable magnesium-ion battery based on a TiSe_2_-cathode with *d*-*p* orbital hybridized electronic structure

**DOI:** 10.1038/srep12486

**Published:** 2015-07-31

**Authors:** Yunpeng Gu, Yukari Katsura, Takafumi Yoshino, Hidenori Takagi, Kouji Taniguchi

**Affiliations:** 1 Department of Advanced Materials Science. The University of Tokyo, 5-1-5 Kashiwanoha, Kashiwa, 277-8561, Japan; 2 Department of Applied Physics. The University of Tokyo, 7-3-1 Hongo, Tokyo, 113-8656, Japan; 3 Department of Physics. The University of Tokyo, 7-3-1 Hongo, Tokyo, 113-0033, Japan; 4 Max Planck Institute for Solid State Research, Heisenbergstrasse 1, Stuttgart, D-70569, Germany; 5 Institute for Materials Research. Tohoku University, 2-1-1 Katahira, Sendai, 980-8577, Japan; 6 Elements Strategy Initiative for Catalysts and Batteries (ESICB). Kyoto University, Katsura, Kyoto, 615-8520, Japan

## Abstract

Rechargeable ion-batteries, in which ions such as Li^+^ carry charges between electrodes, have been contributing to the improvement of power-source performance in a wide variety of mobile electronic devices. Among them, Mg-ion batteries are recently attracting attention due to possible low cost and safety, which are realized by abundant natural resources and stability of Mg in the atmosphere. However, only a few materials have been known to work as rechargeable cathodes for Mg-ion batteries, owing to strong electrostatic interaction between Mg^2+^ and the host lattice. Here we demonstrate rechargeable performance of Mg-ion batteries at ambient temperature by selecting TiSe_2_ as a model cathode by focusing on electronic structure. Charge delocalization of electrons in a metal-ligand unit through *d*-*p* orbital hybridization is suggested as a possible key factor to realize reversible intercalation of Mg^2+^ into TiSe_2_. The viewpoint from the electronic structure proposed in this study might pave a new way to design electrode materials for multivalent-ion batteries.

At present, rechargeable ion batteries have become one of the key systems for energy storage. In particular, lithium-ion batteries have been widely applied for most portable electronic devices as power sources. However, rechargeable batteries of further safety and low cost are recently being required for large scale applications, such as power sources of smart power grids and electric vehicles. Rechargeable magnesium-ion batteries are considered as one potential solution^1^,^2^,^3^,^4^,^5^. Mg-metal, which might be used as a dendrite-free anode^6^,^7^, is stable in ambient atmosphere and abundant in the earth crust compared with Li-metal. In addition, the specific volumetric capacity of Mg reaches 3833 mAh/cm^3^, which is higher than that of Li (2046 mAh/cm^3^), due to the bivalency of Mg-ion.

On the other hand, the development of Mg-ion batteries has been limited because of difficulties in selecting suitable cathode materials. The strong electrostatic interaction between bivalent Mg-ions and host lattices often causes slow solid state diffusion of Mg^2+^ within the local crystal structure and prevents reversible insertion/extraction of Mg^2+^. In the past years, various kinds of materials have been proposed as candidates for cathodes of Mg-ion batteries, such as Chevrel phases Mo_6_*X*_8_ (*X* = S, Se)^8^,^9^, o-Mo_9_Se_11_^10^, TiS_2_ nanotube^11^, graphene-like MoS_2_^12^, WSe_2_ nanowire^13^, Mg*M*SiO_4_ (*M* = Fe, Mn, Co)^14^,^15^,^16^,^17^,^18^,^19^,^20^, MgFePO_4_F^21^, FePO_4_^22^, MnO_2_^23^,^24^, V_2_O_5_ xerogel/thin film^25^,^26^,^27^, organic compounds^28^,^29^,^30^, Prusian Blue analogues^31^,^32^. However, in most cases, Mg-ion batteries could only function by assistance of nanoscale morphology of electrode materials^11^,^12^,^13^,^14^,^15^,^16^,^17^,^18^,^19^,^20^,^21^,^23^,^24^,^25^ or screening of the electrostatic interaction by solvent species such as aqueous ions^31^,^32^. One exception is Chevrel phases, Mo_6_*X*_8_ (*X* = S, Se). Electrochemical performance of micro-crystal Mo_6_*X*_8_-electrodes is reported to be comparable with that of nano-crystal at ambient temperature^9^. In the Chevrel phases, delocalized electronic orbitals of Mo_6_*X*_8_, in which two electrons are accommodated by cluster units of Mo_6_ and the variation of formal charge on individual Mo atom are suppressed to only 1/3*e*, are suggested to possibly moderate local structural deformation induced by the electrostatic interaction between Mg^2+^ and the local lattice, in which extra electrons are accommodated^4^,^33^. As a consequence of charge distribution over multiple atoms, the charge imbalance during the insertion of bivalent ions is supposed to be easily relieved in the Chevrel phase. In the recent studies, other compounds with clustered structures, such as o-Mo_9_Se_11_ and C_60_, are also reported to display reversible Mg-ion insertion reactions^10^,^30^. Thus, systems with delocalized electrons over multiple atoms might be expected as good candidates for rechargeable cathodes of Mg-ion batteries.

In this study, we have focused on the possible effect of electronic delocalization in metal-ligand units through orbital hybridization in transition-metal chalcogenides. When the atomic orbitals hybridize strongly between transition-metals and chalcogens, the electronic wave function of transition-metal chalcogenide spreads on both constituent atoms^34^ (See Figure S1a); The charge density of the introduced electrons should distribute over metal-ligand units as schematically shown in Fig. 1a. On the other hand, in a system with weak orbital hybridization, the electrons should be accommodated only in the transition metal orbitals as displayed in Fig. 1b (See also Figure S1b). In this study, taking into account that the degree of orbital hybridization is enhanced in the case that the difference between energy levels of atomic orbitals is small^35^, layered TiSe_2_ (Fig. 1c) has been selected as a candidate material based on the energy diagram for atomic *d*-orbitals of transition metals and *p*-orbitals of chalcogens (Fig. 1d)^36^. As displayed in Fig. 1d, since the energy levels of valence atomic orbitals in TiSe_2_, 3*d*-orbital of Ti and 4*p*-orbital of Se, are close to each other, the hybridized electronic structure is expected around Fermi energy. In fact, the conducting properties, which could be ascribed to the delocalized electronic structure by orbital hybridization, have been confirmed in TiSe_2_ around room temperature^37^. Here, we report the rechargeable performance of TiSe_2_ with *d*-*p* orbital hybridization as a cathode of Mg-ion batteries.

## Results

**Figure 1:**
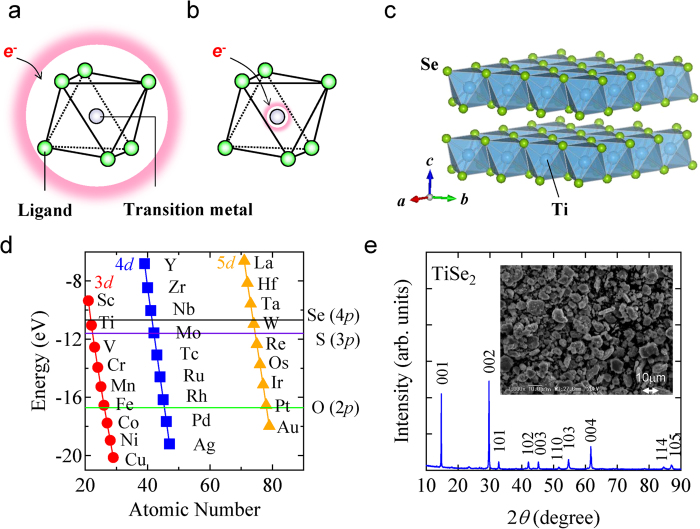
(**a**) A schematic illustration of charge distribution in the electronic state with strong *d*-*p* hybridization. Electrons are accommodated in the delocalized state, which extends over transition metal and ligand atoms as schematically shown by the red framework. (**b**) A schematic drawing of charge distribution in the electronic state with weak *d*-*p* hybridization. Electrons are accommodated only in the localized state of transition metal. (**c**) The crystal structure of 1*T*-TiSe_2_. (**d**) Energy diagram of atomic orbitals (Appendix A in Ref. 36). Red circles, blue squares and orange triangles represent the energy levels of 3*d*-, 4*d*- and 5*d*-orbitals of each transition metal, respectively. The horizontal lines display the energy level position of O 2*p*-orbital, S 3*p*-orbital and Se 4*p*-orbital, respectively. (**e**) The powder X-ray diffraction pattern (XRD) of the TiSe_2_ sample. The inset shows the image of scanning electronic microscopy (SEM) of the TiSe_2_ sample.

The powder X-ray diffraction (XRD) result indicates that TiSe_2_ used in this study takes a 1*T*-type structure (*a* = 3.5393(2) Å and *c* = 6.0103(3) Å) with a space group *P*3-*m*1 (Fig. 1e) as reported in the previous study^38^. As shown in Fig. 1c, the 1*T*-type structure consists of successive Se-Ti-Se sandwich slabs, which are separated by the van der Waals gap. The sample was also checked by Scanning Electronic Microscopy (SEM), showing that TiSe_2_ has a particle size around 10 μm (Fig. 1e).

**Figure 2:**
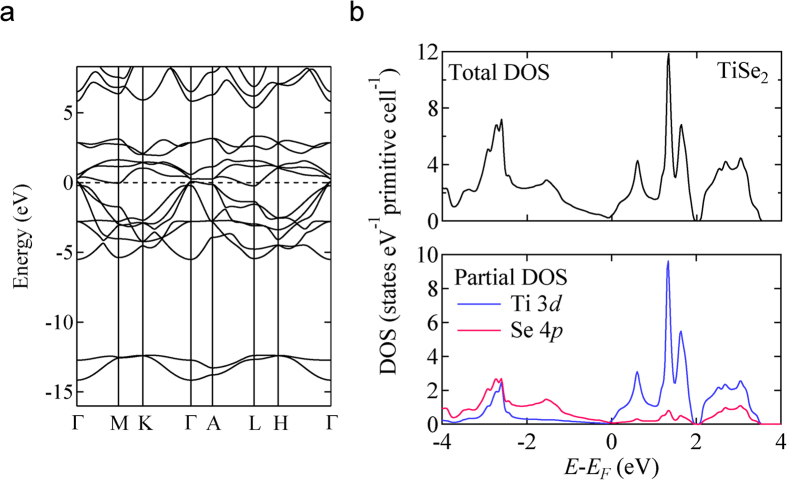
(**a**) Dispersion of the energy band of 1*T*-TiSe_2_. (**b**) Total and partial density of states (DOS) for 1*T*-TiSe_2_.

The orbital hybridization in TiSe_2_ was confirmed by first principle calculation. The energy band dispersion and density of states (DOS) are shown in Fig. 2a,b, respectively. The calculated electronic band structure indicates that 1*T*-TiSe_2_ is a semimetal with a small overlap between the valence band maximum at the Brillouin zone (BZ) center, Γ-point, and the conduction band minimum at the BZ boundary, L-point (Fig. 2a). This electronic band structure is consistent with the previous studies by *Ab initio* band structure calculation^39^ and angle resolved photoemission measurements^40^,^41^. The orbital components of valence and conduction bands indicate the existence of *d*-*p* orbital hybridization. As shown in the partial DOS of TiSe_2_ (see Fig. 2b), the electronic states around the Fermi level (*E*_F_) consist of both 3*d*-orbital of Ti and 4*p*-orbital of Se. In this situation, since the wave function spreads over both Ti- and Se-atoms as shown in Fig. 1a, introduced electrons in the discharge process of TiSe_2_ are expected to be accommodated into a “cluster-like” electronic state.

**Figure 3:**
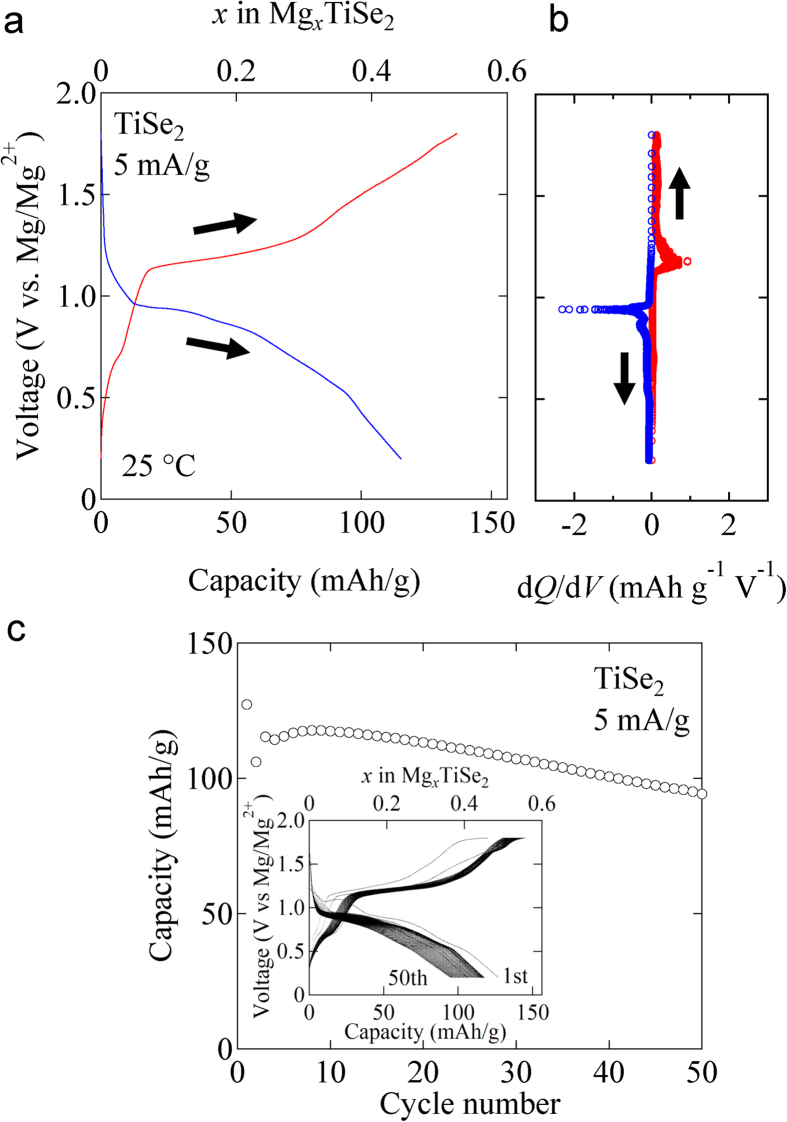
(**a**) The charge/discharge curve (on the second cycle) of the Mg-ion battery cell with TiSe_2_ measured at 25 °C. (**b**) The derivative of the capacity (*Q*) in Fig. 3(a) with respect to the voltage (*V*), d*Q*/d*V*. (**c**) Cycle performance of the Mg-ion battery cell with TiSe_2_ for capacity. The inset shows the charge/discharge curves on each cycle.

The Mg-ion battery with a TiSe_2_ cathode shows reversible electrochemical charge/discharge performance at ambient temperature. Figure 3a displays a typical voltage profile of the coin cell at 25 °C, which consists of a TiSe_2_-based cathode, a magnesium metal anode and 0.25 M Mg(AlCl_2_EtBu)_2_/THF electrolyte solution, in which formation of the passivation film is suppressed thus Mg is reversibly deposited/dissolved on the anode^8^. The derivative of the capacity (*Q*) with respect to the voltage (*V*), d*Q*/d*V*, is plotted in Fig. 3b. The redox peaks are observed in the cathodic process (at 0.9 V vs. Mg/Mg^2+^) and anodic process (at 1.2 V vs. Mg/Mg^2+^), respectively. This result indicates that TiSe_2_ can work as a rechargeable electrode material in the Mg-ion-battery system.

Figure 3c shows cyclability of the TiSe_2_ cathode in the first 50 cycles at 25 °C. The specific capacity is kept at the values around 110 mAh/g during the whole cycling. Assuming that the redox species is Ti^4+^/Ti^3+^, the average specific capacity observed, 108 mAh/g, is about 83% of the theoretical capacity (130 mAh/g). The specific capacity of TiSe_2_ is comparable with that of Mo_6_S_8_ (~100 mAh/g^9^, Chevrel phase), which is the prototype cathode material of Mg-ion batteries.

**Figure 4:**
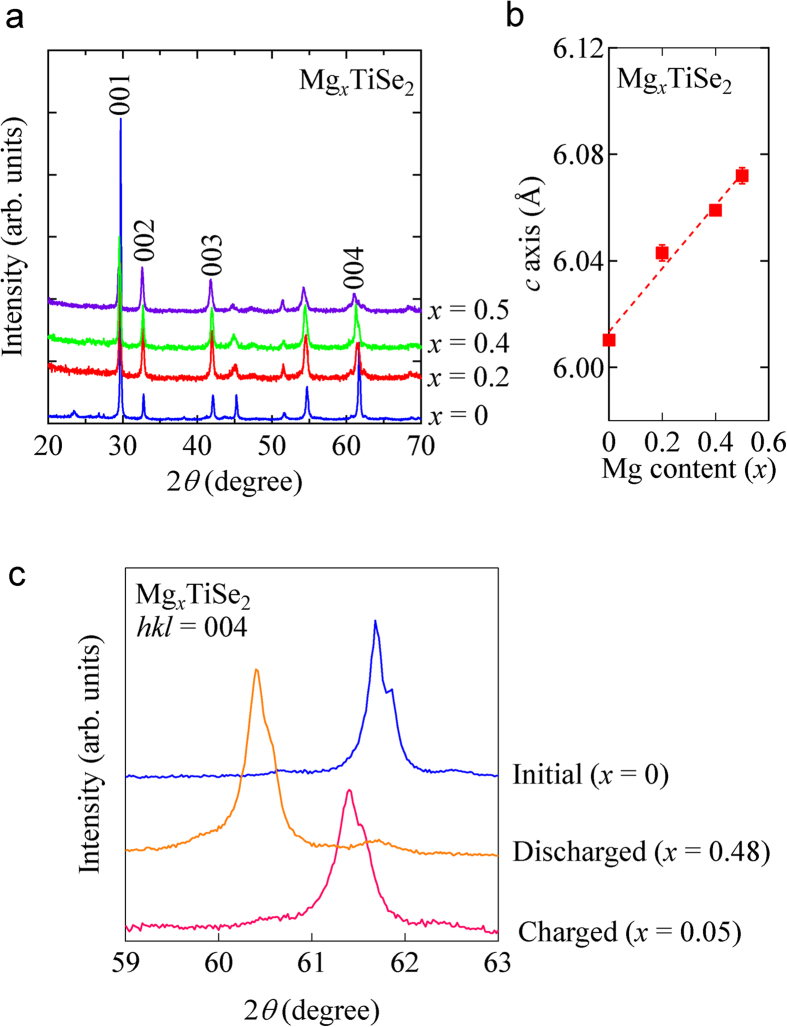
(**a**) Powder XRD of Mg_*x*_TiSe_2_ (*x* = 0, 0.2, 0.4 and 0.5). The *ex situ* XRD measurements were carried out after the discharging processes. (**b**) Mg-content (*x*) dependence of unit cell parameter of *c*-axis. (**c**) 004 peak shift after discharging (*x* = 0.48; orange line) and charging processes (*x* = 0.05; red line). The peak in the initial state (*x* = 0) is represented by the blue line.

Structural variation after the discharge process was traced by measuring *ex situ* XRD patterns of Mg_*x*_TiSe_2_. XRD measurements were performed for selected compositions (*x* = 0, 0.2, 0.4, 0.5). As shown in Fig. 4a, the XRD pattern of TiSe_2_ is maintained after the discharge process. In the XRD pattern of discharged cathode, a small broad peak appears around 47°, which are likely to be assigned to those of Se. Selenium phase seems to be produced by some decomposition of partial cathode, which might be the reason of capacity fading during the cycling. Focusing on the 00*l* peaks (*l* = 1, 2, 3 and 4), all of them gradually shift to the lower angles. This result indicates that Mg^2+^ is intercalated into the van der Waals gap between TiSe_2_ layers in the crystal lattice. During the Mg-ion intercalation, the lattice constant of *c*-axis, which was estimated from the XRD patterns in Fig. 4a, increases from 6.0103(3) Å (*x* = 0) to 6.072(3) Å (*x* = 0.5) (Fig. 4b). The structural reversibility was also confirmed by the *ex situ* XRD measurements. Figure 4c displays the XRD patterns after discharge/charge process. The 004 peak shifts to a lower angle in the discharged state (*x* = 0.48) from its angle in the initial state (*x* = 0), while it shifts back around the initial angle in the charged state (*x* = 0.05). Mg concentrations in TiSe_2_ cathode are confirmed to be *x* = 0.46 for the discharged state (*x* = 0.48 from the electrochemical test) and *x* = 0.12 for the charged state (*x* = 0.05 from the electrochemical test) by inductively coupled plasma atomic emission spectrometry (ICP-AES), respectively. The concomitant variation of Mg concentrations in the discharge/charge process seems to support reversible expansion/contraction picture of the crystal lattice along *c*-axis in TiSe_2_ by intercalation/deintercalation of Mg^2+^ into/from the van der Waals gap.

## Discussion

So far, materials research on cathodes of Mg-ion batteries has been independently performed with emphasis on the crystal structure of each target material in most cases. On the other hand, by introducing the viewpoint based on electronic structures as in this study on TiSe_2_, a common feature seems to appear in the previously reported cathode materials of Mg-ion batteries, such as Mo_6_*X*_8_ (*X* = S, Se)^8^,^9^, TiS_2_^11^, MoS_2_^12^ and WSe_2_^13^. The common feature is that orbital hybridization is expected between the valence atomic orbitals of constituent atoms in these materials, which are *d*-orbital of transition metal atoms and *p*-orbital of chalcogen atoms, due to the close energy levels (Fig. 1d). Intriguingly, such small energy difference between transition metal’s *d*-orbital and ligand’s *p*-orbital can be found not only in the cathodes of Mg-ion batteries but also in the representative ones of Li-ion batteries; Li_*x*_TiS_2_^42^, Li_*x*_CoO_2_^43^ and Li_1-*x*_Mn_2_O_4_^44^ (see Fig. 1d). In these cathode materials of Li-ion batteries, *d*-*p* orbital hybridization can be confirmed in the electronic structures reported by first principle calculations^45^,^46^,^47^. These facts seem to support the efficacy of electron delocalization in metal-ligand units for improving ion-battery performance through *d*-*p* orbital hybridization. In fact, the charge distribution over transition metal and oxygen atoms are pointed out for Li(Co, Al)O_2_ by first principle calculation^48^ and are confirmed in Li_*x*_CoO_2_ and Li_*x*_Mn_2_O_4_ by X-ray spectroscopy^49^,^50^.

In the case of selenides, *d*-*p* orbital hybridization should be enhanced by high orbitals overlap due to the large 4*p*-orbital size of Se, compared with oxides or sulfides. Additionally, in TiSe_2_, the two-dimensionality of ion conducting channels in the van der Waals gap might contribute to suppress the effect of Coulomb repulsion between Mg-ions^10^. These factors in the TiSe_2_ cathode might contribute to its reversibility on electrochemical cycling and high specific capacity close to the theoretical value in the Mg-ion battery even with micro-sized electrode. In fact, the Mg-ion battery with another cathode material, 1*T*-VSe_2_, where the electronic and crystal structures are similar with those of 1*T*-TiSe_2_, also shows rechargeable cathode performance over 60 cycles with kept relatively high capacity of ~110 mAh/g at ambient temperature (Figure S2a, b). In these materials (TiSe_2_ and VSe_2_) that can work as reversible Mg-ion battery cathodes in the form of micro-sized particles, further improvement in cyclability and rate capability might be expected by downsizing cathode materials to the nanometric scale as done in graphene-like MoS_2_^12^ and WSe_2_ nanowire^13^.

The viewpoint from electronic structure of cathode materials could draw a guideline for developing high voltage Mg-ion batteries. In general, open circuit voltage of an ion-battery (*V*_oc_) is given by difference in electrochemical potentials of electrons between anode (*μ*_A_^e^) and cathode (*μ*_C_^e^); *eV*_oc_ = *μ*_A_^e^ – *μ*_C_^e^, where *e* is magnitude of electron charge^51^,^52^. Taking into account that the electrochemical potential of electrons is defined by Fermi level in vacuum and inner-potential, trend of ion-battery voltage could be assessed by difference in Fermi levels between two electrodes in first approximation. Since the Fermi level of cathode is determined by the electronic structure of valence/conduction band, the voltage of battery using same anodes should reflect the energy levels of valence atomic orbitals, which constitute a valence/conduction band. In the case of TiSe_2_, because the energy levels of Ti 3*d*-orbital and Se 4*p*-orbital are located in the shallow energy range compared with late transition metals and oxygen as shown in Fig. 1d, the observed voltage, ~1 V vs. Mg/Mg^2+^, seems to be a lower value than those of Li-ion batteries with transition metal oxides, such as Li_*x*_CoO_2_ and Li_1-*x*_Mn_2_O_4_. In further material research for Mg-ion battery cathodes with improved performance, late transition metal oxides might be good candidates for reaching high voltages cells, because their valence bands consist of atomic orbitals located in the deep energy range. In addition, since selenium is a rare element that may be toxic, developing cathodes of oxides could be desirable from the standpoint of practical use and environmental concerns.

In summary, this study has demonstrated that the Mg-ion battery with a micro-sized TiSe_2_ cathode shows rechargeable performance at ambient temperature. In TiSe_2_, reflecting the close energy levels of 3*d*-orbital of Ti and 4*p*-orbital of Se, *d*-*p* orbital hybridization around the Fermi level was confirmed by first principle calculation. The XRD measurements indicate that Mg-ions are reversibly intercalated into and deintercalated from the van der Waals gap between the TiSe_2_ layers. We have proposed one possible scenario that the charge delocalization in metal-ligand units by strong *d-p* orbital hybridization might be one of the key factors, which improve reversible performance of Mg^2+^-intercalation/deintercalation. Further study based on the electronic structure could open a new way to design cathode materials for Mg-ion and other multivalent ion batteries.

## Methods

### Sample preparation and electrochemical measurements

The purchased TiSe_2_ powder (>99%, High Purity Chemicals) was used as a cathode material. Cathodes were fabricated using TiSe_2_, acetylene black (AB) and polytetrafluoroethylene (PTFE) in a mass ratio of 81:9:10. The mixed active material was pressed on a current collector of copper net and dried at 60 °C in vacuum. The amount of active material was approximately 3 ~ 4 mg per cathode. Tests of Mg-ion batteries were performed by 2032-type coin cells, which were assembled in an Ar-filled glove box. These cells consisted of TiSe_2_ cathodes, magnesium metal anodes and the Mg(AlCl_2_EtBu)_2_ electrolyte dissolved at 0.25 mol/dm^3^ in tetrahydrofuran (THF). The galvanostatic charge/discharge tests were conducted using a potentio-galvanostat (Solartron, 1470E) at 25 °C. Current density dependence of capacity was tested for 5 mA/g, 10 mA/g, 20 mA/g and 50 mA/g, respectively (Fig. S3(a)). Rate capability, which is defined as the capacity ratio to the discharge capacity at 5 mA/g, decreases to *ca*. 50% at high current density of 50 mA/g (see Fig. S3(b)). The cell voltage in the galvanostatic charge/discharge measurements was cycled between 1.8 V and 0.2 V (vs. Mg/Mg^2+^) for 50 times by applying a constant current at 5 mA/g. Electrochemical characteristic of TiSe_2_ in a electrolyte solution other than Mg(AlCl_2_EtBu)_2_ /THF was examined through Cyclic voltammetry (CV) measurements with a three-electrode cell. As an electrolyte for CV measurements, Mg(ClO_4_)_2_ was dissolved in acetonitorile (AN) at 1 mol/dm^3^. A magnesium metal and a silver wire, which was put into a solution of 0.01 mol/dm^3^ AgNO_3_ and 0.1 mol/dm^3^ tetrabutylammonium perchlorate in acetonitorile, were employed as a counter electrode and a reference electrode, respectively. In the CV measurement, the potential was scanned between 0 V and –1.5 V (vs. Ag/Ag^+^) at 0.5 mV/s. In the voltammogram, one peak was clearly observed for both cathodic and anodic processes, respectively (Fig. S4).

### Material characterization

Structural characterization of samples was performed to analyze the change of TiSe_2_ upon cycling by *ex situ* powder X-ray diffraction (XRD) using a graphite monochromatized Cu Kα radiation source. The particle size of sample was measured by scanning electronic microscopy (SEM). The variation of the Mg concentration in the TiSe_2_ cathodes after discharge/charge process was traced by quantitative chemical analysis. The atomic ratio of Mg to Ti was determined quantitatively by inductively coupled plasma atomic emission spectrometry (ICP-AES). The electrodes were removed from the cells after the charge/discharge cycling process and rinsed with THF to dissolve out the electrolyte salt in an Ar-filled glove box before the measurements. In the *ex situ* structural analysis of electrodes, electrochemically prepared electrode samples were measured immediately after being transferred out of the glove box, or sealed with polyethylene-nylon films in an Ar-filled glove box before XRD measurements.

### First-principle calculation

First-principle electronic structure calculations were performed by Full-potential Linearized Augmented Plane Wave (FLAPW) method implemented in WIEN2k^53^ code, under Tran-Blaha modified Becke-Johnson (TB-mBJ) exchange-correlation potential^54^. Muffin-tin radii for Ti and Se were set to 2.50 and 2.25 a.u., respectively. The first Brillouin zone was divided into 21 × 21 × 10 *k*-point mesh with 436 irreducible points. No spin polarization was considered. Obtained density of states (DOS) was rescaled to the primitive unit cell.

## Additional Information

**How to cite this article**: Gu, Y. *et al.* Rechargeable magnesium-ion battery based on a TiSe_2_-cathode with *d*-*p* orbital hybridized electronic structure. *Sci. Rep.* **5**, 12486; doi: 10.1038/srep12486 (2015).

## Supplementary Material

Supplementary Information
