# Supplementary material for: Rechargeable magnesium-ion battery based on a TiSe2-cathode with d-p orbital hybridized electronic structure
Source: Sci Rep. 2015 Jul 31;5:12486. doi: 10.1038/srep12486 (PMC4521182; doi:10.1038/srep12486)
Supplement: Supplementary Information [file srep12486-s1.pdf]

Rechargeable magnesium-ion battery based on a $TiSe_2$-cathode with *d-p* orbital hybridized electronic structure

Yunpeng Gu[1], Yukari Katsura[2], Takafumi Yoshino[1], Hidenori Takagi[1,3,4] and Kouji Taniguchi*[5,6]

[1]Department of Advanced Materials Science
The University of Tokyo, 5-1-5 Kashiwanoha, Kashiwa, 277-8561, Japan.

[2]Department of Applied Physics
The University of Tokyo, 7-3-1 Hongo, Tokyo, 113-8656, Japan.

[3]Department of Physics
The University of Tokyo, 7-3-1 Hongo, Tokyo, 113-0033, Japan.

[4]Max Planck Institute for Solid State Research,
Heisenbergstrasse 1, Stuttgart, D-70569, Germany.

[5]Institute for Materials Research
Tohoku University, 2-1-1 Katahira, Sendai, 980-8577, Japan.

[6]Elements Strategy Initiative for Catalysts and Batteries (ESICB)
Kyoto University, Katsura, Kyoto, 615-8520, Japan.

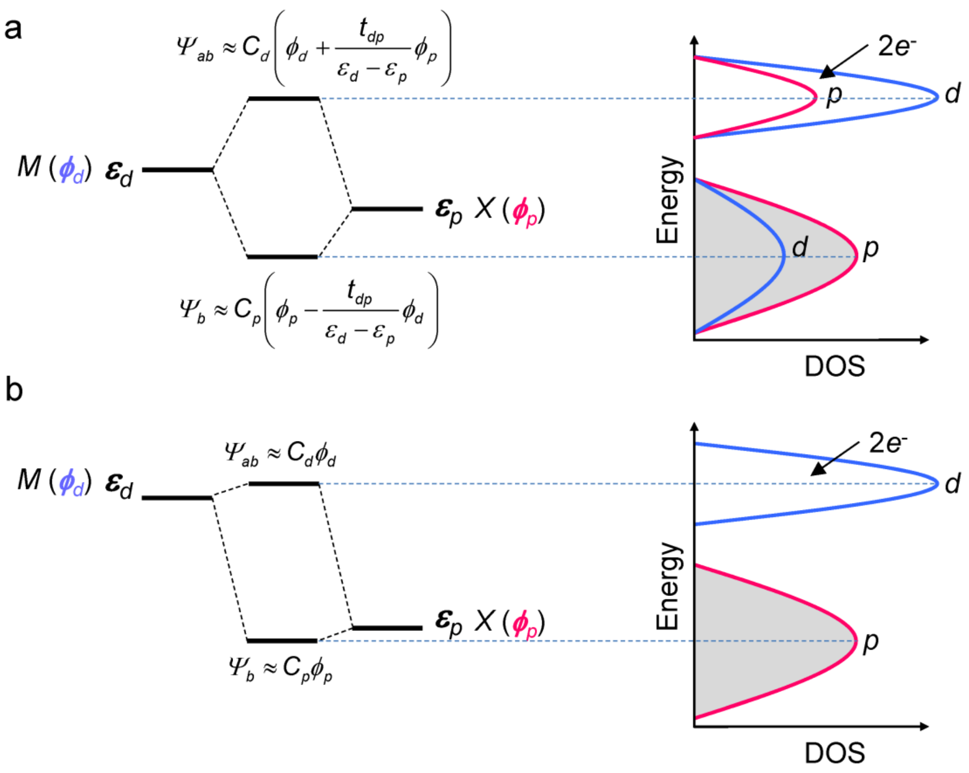

Figure S1: Schematic picture of *d-p* orbital hybridization in transition metal chalcogenide Illustration of molecular orbitals formed by local interaction (t_{dp}) between transition metal (*M*) *d*-orbital (ϕ_d) and ligand (*X*) *p*-orbital (ϕ_p), with their partial DOS consisting of valence and conduction bands. ε_d and ε_p are energy levels of ϕ_d and ϕ_p, respectively. Ψ_b and Ψ_{ab} are the bonding and antibonding molecular orbitals. In transition metal chalcogenides, Ψ_b are fully occupied by electrons, and the introduced electrons are accommodated in Ψ_{ab}. Shaded areas in DOS are occupied by electrons. (a) Molecular orbitals formed by strong *d-p* orbital hybridization. Orbital hybridization is enhanced when ε_d and ε_p are close to each other. Molecular orbitals consist of both ϕ_d and ϕ_p. As a result, conduction/valence band is composed of both transition metal *d*-orbital and ligand *p*-orbital. (b) Molecular orbitals formed by weak *d-p* orbital hybridization. Reflecting large energy difference between ε_d and ε_p, each molecular orbital consists of only ϕ_d or ϕ_p. In this situation, an ionic picture is suitable, and the characteristic of conduction band (valence band) is well described by only *d*-orbital of transition metal (*p*-orbital of ligand).

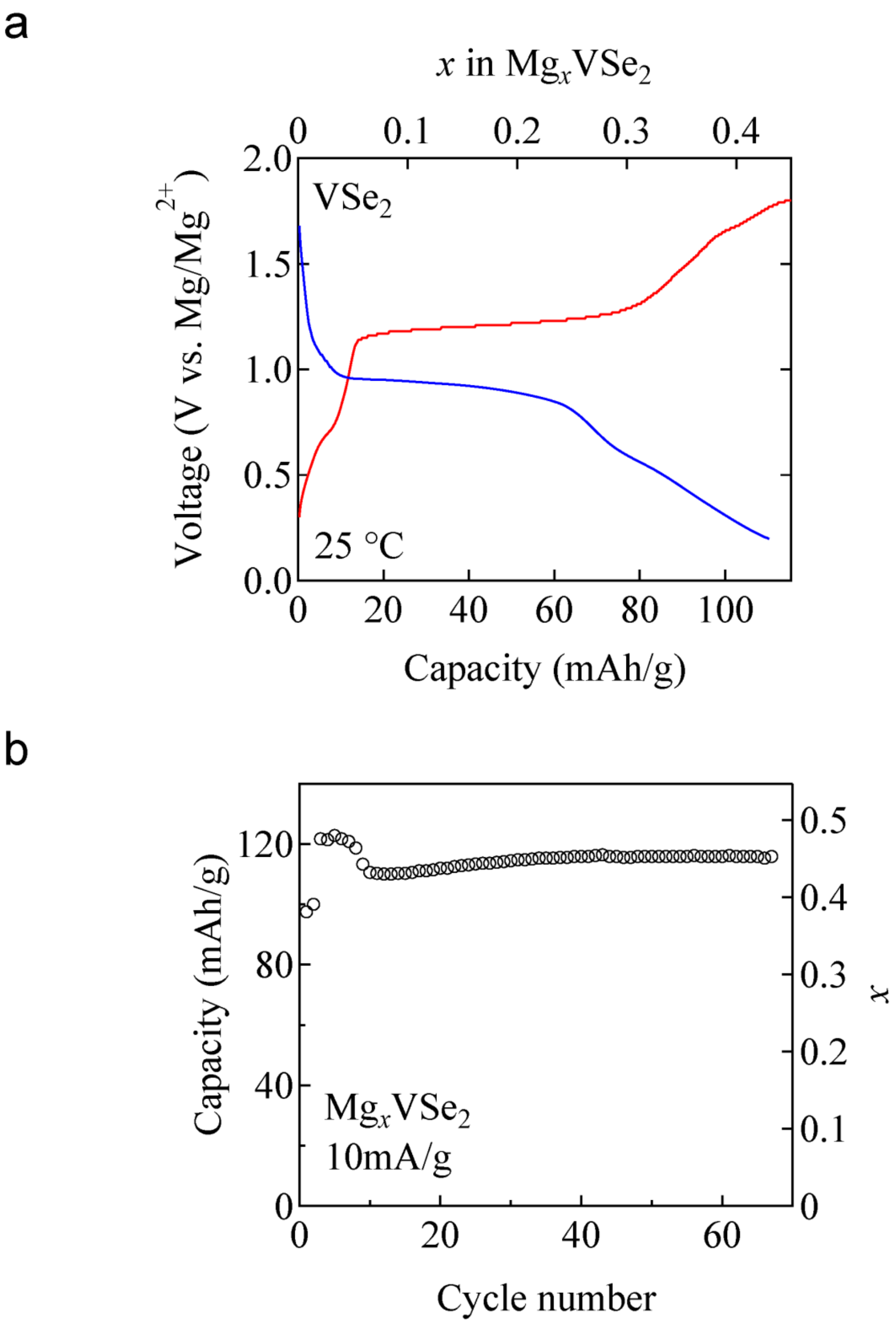

Figure S2: (a) The charge/discharge curve (on second cycle) of Mg-ion battery cell with VSe_2 cathode measured at 25 °C. (b) Cycle performance of Mg-ion battery cell with VSe_2 for capacity.

a

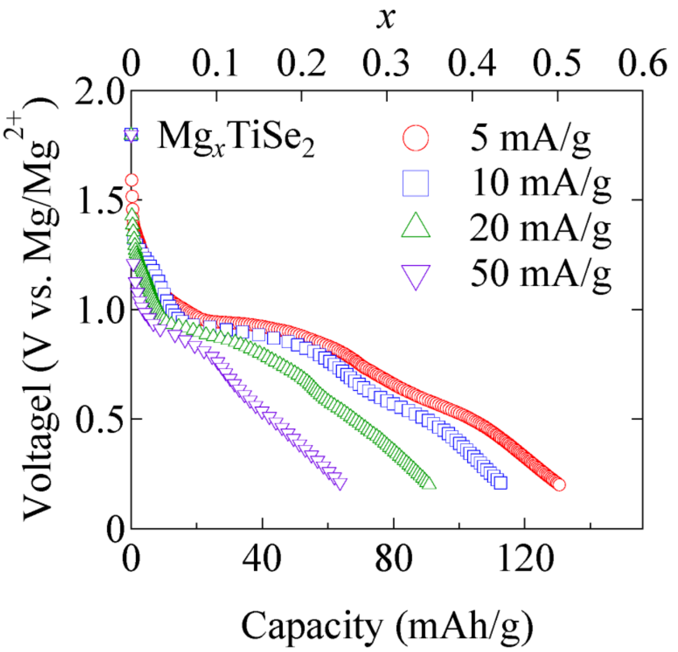

b

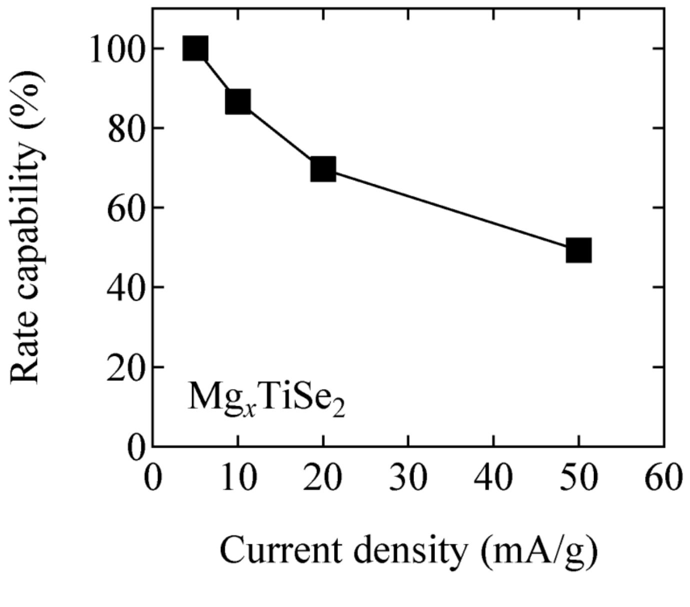

Figure S3: (a) The current density dependence of discharge curve (on second cycle) of Mg-ion battery cell with $TiSe_2$ cathode. (b) Rate capability of Mg-ion battery cell with $TiSe_2$ cathode.

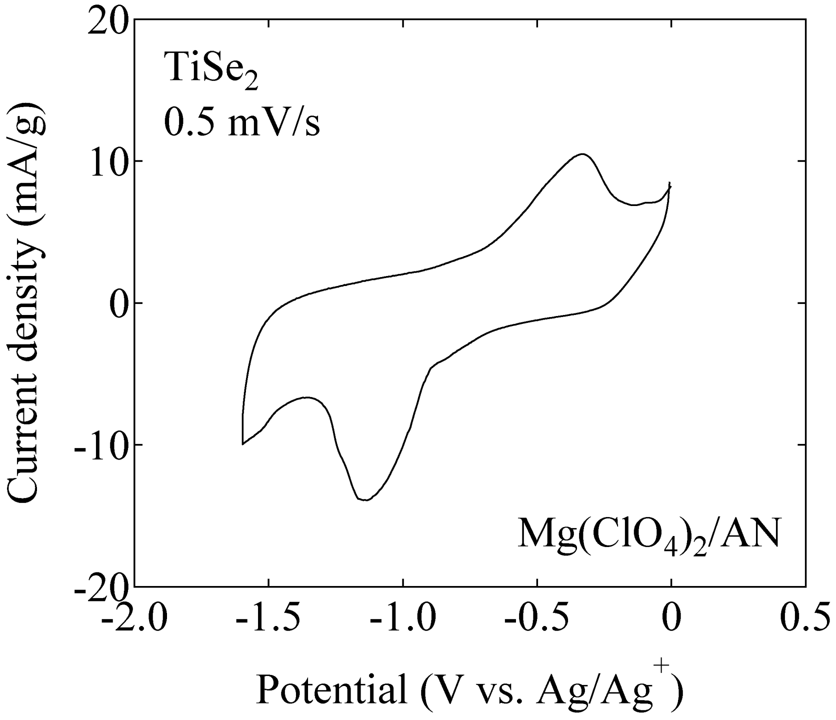

Figure S4: The cyclic voltammogram of $TiSe_2$ electrode in $Mg(ClO_4)_2$/AN at a sweep rate of 0.5 mV/s.
